# Smartphone movement data can reliably predict smoking lapses and cravings to enable timely smoking cessation support

**DOI:** 10.1038/s41598-026-49611-y

**Published:** 2026-05-21

**Authors:** Maryam Abo-Tabik, Nicholas Costen, Yael Benn

**Affiliations:** 1 Department of Computer Science, University of Central Lancashire, Preston, UK; 2 Department of Computing and Mathematics, Manchester Metropolitan University, Manchester, UK; 3 School of Psychology, Manchester Metropolitan University, Manchester, UK

**Keywords:** Computer science, Predictive markers, Predictive markers

## Abstract

Decades of research aiming to develop effective smoking interventions have identified triggers that contribute to failed quitting attempts including environmental (e.g. location), social (presence of other smokers), or internal (e.g. stress). Here, it is shown for the first time that passively collected movement data from smokers’ smartphones’ sensors (accelerometer, gyroscope and magnetometer) can be used to predict smoking-behaviour. Feeding the movement data into a Deep Learning (DL) model (1D-CNN-BiLSTM), smoking-behaviour was predicted with 85% accuracy within the subsequent 5-minute window. This compares to 63% accuracy when using traditional triggers (e.g. time of the day). Crucially, movement data can be used to predict high-craving incidents and lapses in the 3 months period following quitting smoking with similarly high accuracy, even when predictions are made without any personal data (i.e. when the model is trained using only data from other smokers). These findings can transform smoking-cessation apps, enabling the provision of just-in-time personalised support to those wishing to quit smoking. Importantly, the findings have implications beyond smoking-cessation applications, by revealing that human movements, largely overlooked to date, can be used for early detection of, and intervention for, health (and other) behaviours, including those that are not genetic or typically characterised by changes in movement.

## Introduction

Smoking is a public health emergency, increasing the risks for multiple health conditions, which result in the death of around 8 million people worldwide annually^1,2^. While many smokers try to give up smoking, quitting success rate remains low^3^. To support smokers who wish to quit, many mobile-based smoking cessation apps have been developed^4^. While there are several popular applications that are effective in improving quitting rate (for example, the United Kingdom National Health Service quit smoking app^5^), these apps do not make use of the full range of the currently available technology^4^, and hence provide less than optimal support.

Studies indicate that providing just-in-time targeted intervention can minimise relapse incidents^6,7^. To enable the delivery of such timely intervention (e.g. via a mobile app), several attempts have been made to utilise Machine Learning (ML) algorithms for predicting smoking-behaviour based on known smoking risk-factors such as high urges^8^, geographical location^9^ or social triggers^10^. However, these studies suffer from several limitations. For one, the data they are based on largely relies on self-reporting from smokers (e.g. 8), which is considered unreliable, as self-reporting is often affected by recall issues, social bias and low commitment, leading to potentially misleading results in health outcomes and behaviour assessments^11^.

Multiple mobile sensors such as accelerometers or Global Positioning System (GPS) are now commonly embedded within smartphones and smartwatches, and they offer a superior method for passively collecting real-time and objective data. Such data have increasingly been used within ML algorithms to analyse and understand complex behavioural patterns to improve insight into different behaviours and medical conditions^12^.

Recent studies have tended to use GPS signals to predict smoking events. Abo-Tabik et al.^13^ fed passively collected GPS and accelerometer data from smartphones, through a combined 1D Convolutional Neural Network (1D-CNN) and Control Theory model. While the model achieved 0.74 accuracy, it was trained only on regular, pre-quit smoking data, meaning its ability to predict lapses after quitting remains unclear. Furthermore, the model was restricted to predicting events within a 60-minute window, limiting its utility for “just-in-time” interventions. In addition to the specifics of the model, using GPS data has several notable limitations. First, GPS data are highly personalised, which reduces generalisability because models trained on one participant’s data cannot readily be applied to others. Second, GPS collection raises privacy and security concerns due to sensitive location information, meaning many phones operating systems now restrict its collection. Lastly, GPS is power-intensive because it must receive signals from multiple satellites and perform computationally expensive trilateration^14^, meaning it drains the phone’s battery. By contrast, focusing on high-frequency inertial sensor data (e.g. accelerometers) provide less intrusive, privacy-preserving input that is more generalisable and well-suited for continuous monitoring.

Movement data from smartphones and wearable sensors have been previously used by researchers to study and predict smoking behaviour^15^. Findings suggest that while wearable motion sensors can accurately detect hand-to-mouth gestures in labs, they often perform worse in real-life; possibly due to user behaviour or the need for exact location of device placement (e.g. on the arm). Combining data from smartwatch and a custom wearable finger sensor revealed promising results for detecting smoking-related actions^14^ (much better than smartwatch alone), however, while relying on wearing multiple devices may be good in lab settings, it is unlikely to work in real-life conditions.

Given the increase in smartphones ownership, their improved computing power, and the multiple sensors, such as the accelerometer and GPS, that are now commonly embedded within smartphones, they offer a superior method for passively collecting real-time and objective data. This is particularly attractive if the data from smartphones could be collected in Naturalistic and unconstraint settings. In such settings, the device is not required to be attached to the body, nor is specific training required. Such data have increasingly been used within ML algorithms to analyse and understand complex behavioural patterns to improve insight into different behaviours and medical conditions^11^.

Recent studies have begun to use movement data alone, collected by digital sensors, to predict and understand behaviours in both humans (for example, disruptive behaviour among children (16); calssification of stress or anxietyindicators (17)), and animals (e.g. Ref.^18^). Furthermore, recent work had shown that some micro-movements, which even when detectable to the human eye often go unnoticed (e.g. pupil and head movement), can be used effectively to diagnose conditions such as Autism^19^. The method, currently referred to as ‘Behavioural Phenotyping’, has the potential to target a wide range of health behaviours^20–22^, but to date has not been used to identify or characterise non-genetic conditions. The integration of sensor technology with Artificial Intelligence (AI) has opened new opportunities for digital phenotyping and health monitoring, enabling the passive collection of sensors’ data, call history, SMS patterns, and application usage, to be utilised for behavioural characterisation and prediction^23^. Here, the question is whether passively collected smartphone sensor data (rather than body-attached wearable sensors), processed through DL techniques, can be used to accurately predict smoking-related behaviour prior to and during a quit attempt.

Thus the aim is to identify a suitable DL model that can reliably predict smoking events within a 5-minutes window, based on data collected from unrestricted use of mobile phone. A five-minutes period is a suitable period for applying a just-in-time intervention. To this end, this paper reports the outcome of a two-part study that used sensor data, collected from smokers’ smartphones during unrestricted use, to predict smoking-behaviour before and after quitting. The outcomes demonstrate the potential of human movements, as detected by digital sensors but perhaps not consciously registered by humans, to identify seemingly unrelated behaviours or conditions. As such, the technology offers opportunities for characterising a wide-range of behaviours, specifically supporting early detection and intervention for problematic health behaviours.

## Results

Findings derive from analysis of data collected from 17 smokers, over 3.5 months, using a simple smartphone app. In Phase 1 (two weeks) of the data collection smokers were asked to report in the app every cigarette they smoked. Phase 2 (3 months) took place once the smoker quit smoking (no more than 5 days after Phase 1 ended). In phase 2, participants were asked to report any smoking lapses. They were also asked to report their craving level on a scale from 1 (very low) to 5 (very high), every time that they experienced high craving, or at least once a day (whenever they remembered to do so). Smartphones’ sensor data (Accelerometer: ACC; Gyroscope: GYR; Magnetometer: MAG; Light: L; Time of the Day: T and GPS) were continuously recorded every minute throughout both phases of data collection. Since there has been no previous research conducted in similar settings, project evaluation was carried out by comparing multiple DL architectures across various experimental conditions, as described below.

### Using each smoker’s Phase 1 data to predict smoking-behaviour (in Phase 1), and lapses and cravings (in Phase 2)

At the first stage, the performance of four DL models was evaluated: 1D-CNN, Long Short Term Memory (LSTM), Bidirectional LSTM (BiLSTM), and 1D-CNN-BiLSTM. These DL models were selected based on their validated ability to extract feature patterns from streaming raw sensor data and their demonstrated effective results in predicting behaviour across a range of other applications. The evaluation here focused on addressing the study aim, i.e. assessing the capability of DL models to perform behavioural phenotyping using data passively collected from smartphone sensors, without imposing any restrictions or constraints on users as they engaged in their daily activities. To this end, The DL models were tested for their ability to predict smoking events using smokers’ Phase 1 data (pre-quit data). The data used was non-overlapping 5-minute sliced windows for each participant, which were first shuffled to avoid temporal data leakage, then utilising 90% of each smoker’s collected Phase 1 data to training-validation the model, in order to predict smoking events in the remaining 10% of the Phase 1 ’test’ data.

**Table 1:** Comparison of models’ accuracy in predicting smoking events pre-quitting (Phase 1), and lapses and cravings post-quitting (Phase 2).

Input type	LSTM	1D-CNN	BiLSTM	1D- CNN-BiLSTM
(A) Individual smokers’ Phase 1 data, used for predicting their smoking events				
Accelerometer (ACC)	0.833	0.818	0.841	0.847
Gyroscope (GYR)	0.829	0.818	0.826	0.826
Magnetometer (MAG)	0.550	0.771	0.585	0.795
Light (L)	0.702	0.756	0.698	0.752
Time of the Day (T)	0.632	0.651	0.632	0.632
ACC-GYR-MAG	0.574	0.798	0.554	**0.854**
(B) Individual smokers’ Phase 1 data, used for predicting their lapsing and craving at Phase 2				
Accelerometer (ACC)	0.767	0.743	0.735	0.772
Gyroscope (GYR)	0.755	0.753	0.769	0.759
Magnetometer (MAG)	0.459	0.717	0.503	0.734
Light (L)	0.670	0.665	0.667	0.683
Time of the Day (T)	0.603	0.596	0.603	0.602
ACC-GYR-MAG	0.509	0.713	0.517	**0.775**

Best prediction value in each section is indicated in bold.

Table 1(A) displays the prediction accuracy of the different models using different sensor data as input. The table reveals that the combined 1D-CNN-BilSTM can predict smoking events with 0.85 accuracy within a 5-minute window, using the phone’s movement data alone (ACC, GYR and MAG). Subsequently, the same DL models were employed, trained with 100% smokers’ Phase 1 data, to test their capacity in predicting smokers’ Phase 2 (12-week post-quit period) smoking lapses and reported cravings (Table 1(B)). This approach is important for both demonstrating the capacity of the model to predict lapses, and for minimisng potential effect of temporal leakage. Again, the 1D-CNN-BilSTM was superior, predicting behaviour with 0.78 accuracy within a 5 minute window.

It is note-worthy here that data from sensors previously not considered by scientists for characterising smoking behaviour, i.e. movement data, have very high predictive value (e.g. ACC, 0.77). This is in contrast to data such as, “time of the day”, which was previously considered a major predictive value for smoking-behaviour^23,24^, but in fact provides the lowest single predictive value (0.60). However, the relatively low predictability of time and light sensors could be due to the nature of the data, which can cause class overlapping. The predictive value of the MAG sensor may suggest that the model is relying on magnetic data that is associated with specific locations of the phone. However, although MAG alone achieved an accuracy of 0.795 in Phase 1 and 0.734 in Phase 2, it was not the most informative modality when compared with GYR and ACC. Furthermore, incorporating MAG alongside ACC and GYR resulted in only a marginal improvement in performance (0.847 to 0.854) in Phase 1, and (0.772 to 0.775) in Phase 2. It is also important to note here again that data collection was conducted under naturalistic, real-world conditions in which participants used the application without any constraints on device placement. This approach introduced substantial variability in sensor orientation, location, and surrounding physical environments. Such diversity reduces the likelihood that the model is leveraging location-specific magnetic signatures or environmental artifacts. Instead, the observed performance suggests that the model primarily captures generalisable patterns of human movement rather than environment-dependent cues. This is further illustrated in the section below:Using other smokers’ Phase 1 data, to predict Phase 2 lapses and cravings of a new smoker, where it is shown that the model is able to predict behaviour using data collected from other smokers.

**Fig. 1:**
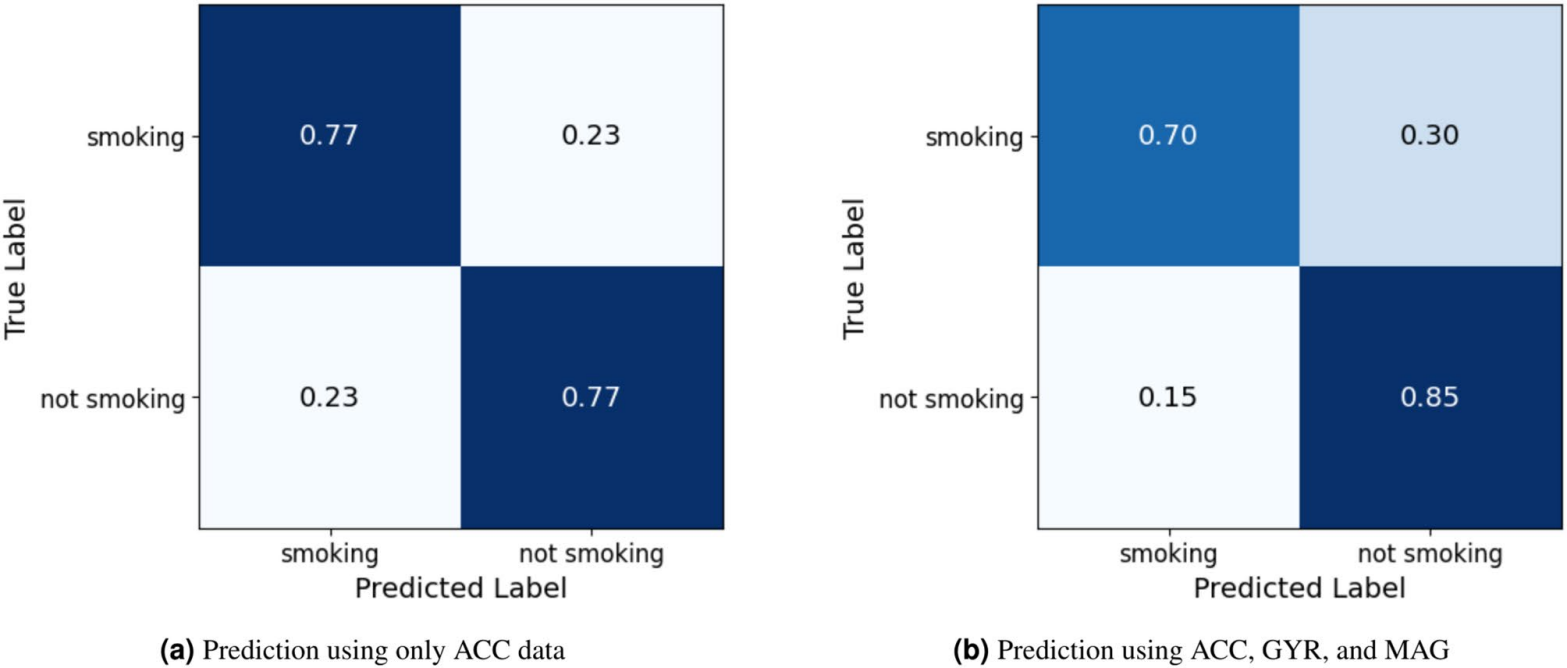
1D-CNN-BiLSTM confusion matrix.

The confusion matrix (Fig. 1), reveals a high 0.85 accuracy when all three sensors are used, compared to lower accuracy by each sensors separately. This difference is critical when implementing smoking interventions, to avoid sending false intervention-messages to smokers, which may, instead of helping, act as a reminder of their smoking habit, with negative impact on their quitting attempt^9^. For this same reason, some prefer to report the positive and negative predictive values (PPV and NPV respectively)^25^, which is reported in Appendix A, but overall accuracy was chosen as the investigators believe that this best represents the potential efficiency of intervention, delivering intervention only at the right time.

To validate the efficiency of the algorithm for use within smart smoking cessation apps, a more in-depth analysis of the model’s ability to differentiate between lapses and craving events was conducted. Phase 2 labels were reclassified into either ‘smoking’ (actual lapse) or ‘craving’ (every instance when the smoker reported a craving level ranging from 1 to 5). The rationale is that even if reported craving is low, the act of reporting indicates that the smoker had thought about their smoking habit at that moment. In both the above analyses the overall accuracy of the integrated 1D-CNN & BiLSTM model shows minimal difference in performance when using ACC data alone as input compared to using the combined input from ACC, GYR, and MAG sensors. However, in both cases the 1D-CNN-BiLSTM model demonstrates enhanced ability in predicting smoking events, lapses and reported cravings when data from all three movement sensor types are combined.

**Fig. 2:**
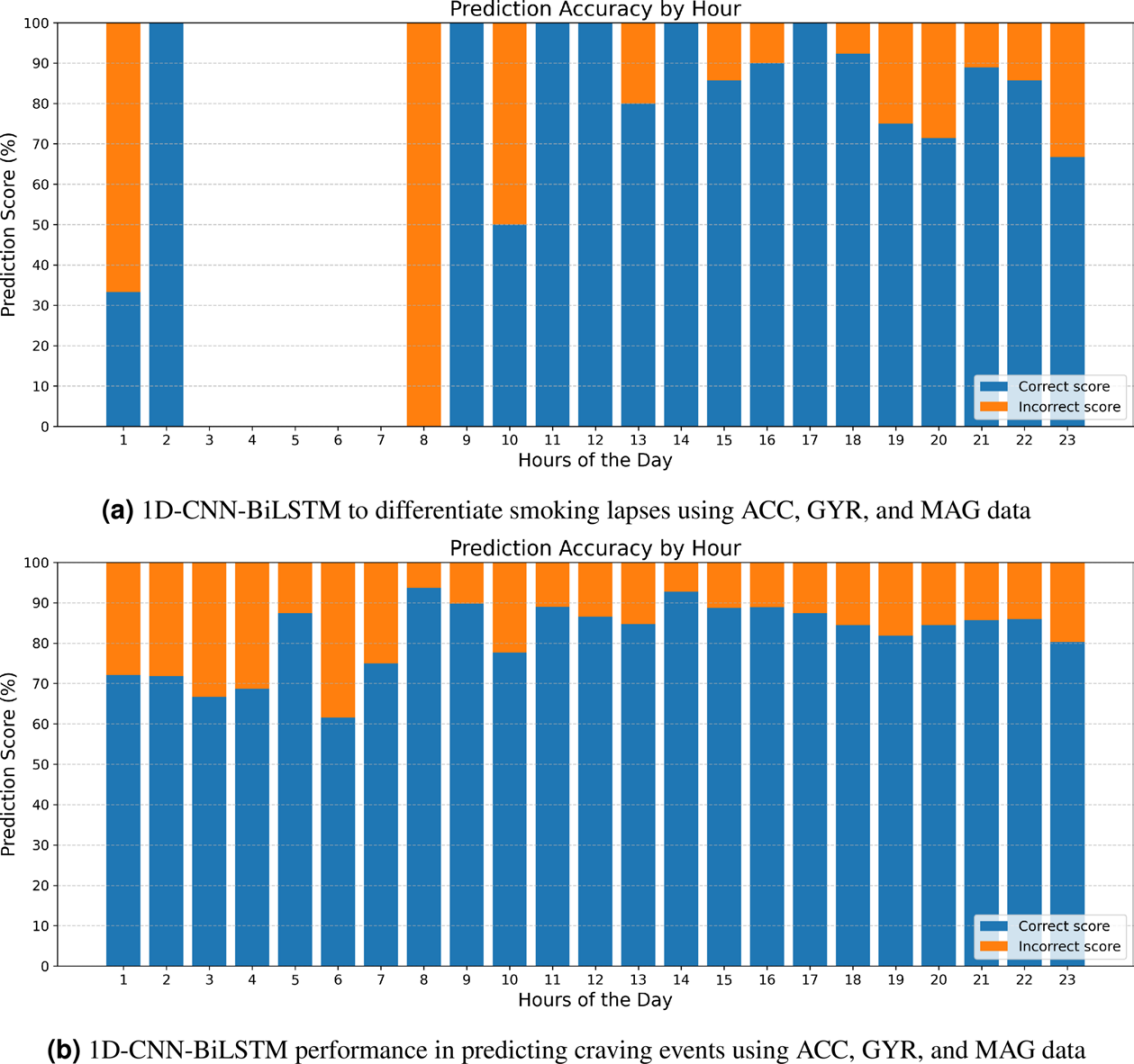
1D-CNN-BiLSTM prediction score for smoking lapses and craving during different hours of the day.

Figure 2 shows the efficiency of the combined ACC, GYR and MAG inputs in predicting moments of either smoking lapse (a) or craving-reporting (b) at different time of the day.

**Fig. 3:**
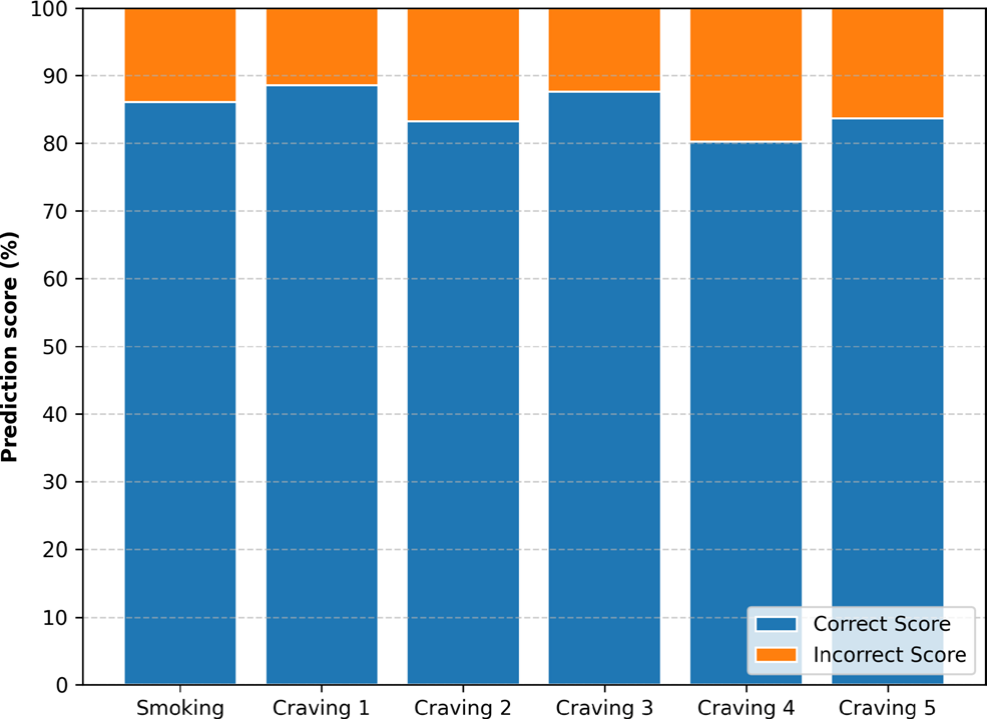
Smoking lapses and self-reported craving level (on a scale from 1-5) predictions as made by the 1D-CNN-BiLSTM model with input from ACC, GYR, and MAG sensors.

Lastly, Fig. 3 shows the model’s prediction accuracy for cravings at various reported levels (1, 2, 3, 4, or 5), along with smoking lapses. The results clearly demonstrate the ability of the model to recognise and differentiate lapse incidents and reported craving events. This highlights the potential of the model for supporting individuals through the challenges of quitting their smoking addiction by effectively intervening in timely manner during high-risk scenarios associated with lapsing.

### Using other smokers’ Phase 1 data, to predict Phase 2 lapses and cravings of a new smoker

If the model is able to predict lapsing behaviour of any smoker using a model trained by the smoking and lapse data collected from other smokers, it would mean that an app can be downloaded by smokers and instantly used to support their quitting, without having to go through the two-week ‘training’ period to learn the individual’s pattern. It would also mean that smokers’ movements, as recognised by their smartphone’s sensors, are universal in the identification of smoking lapses. This would suggest that those movements, which have not to date been identified by scientists despite decades of research into smoking behaviour, may offer a window to the diagnosis or intervention of other conditions or behaviours.

A leave-one-participant-out (LOPO) validation process demonstrates that craving and lapse behaviours can be predicted in previously unseen individuals. This approach also avoids overfitting and ensures generalisability. If prediction accuracy is high, it will mean that the detected micro-movement patterns are generalisable across smokers, rather than being specific to a particular participant. To test the accuracy of the DL model in making such predictions, each participant’s Phase 1 data was, in turn, held back from the training set, and the model was tested using the Phase 2 data of that participant. In this test only 14 out of the 17 participants’ data was used because three participants did not report any post-quitting data. Only the 1D-CNN-BiLSTM with 9-vector inputs from the ACC (x,y,z), GYR (x,y,z) and MAG (x,y,z), was used, given the superiority of the model in predicting smoking and lapses in the previous analysis.

**Fig. 4:**
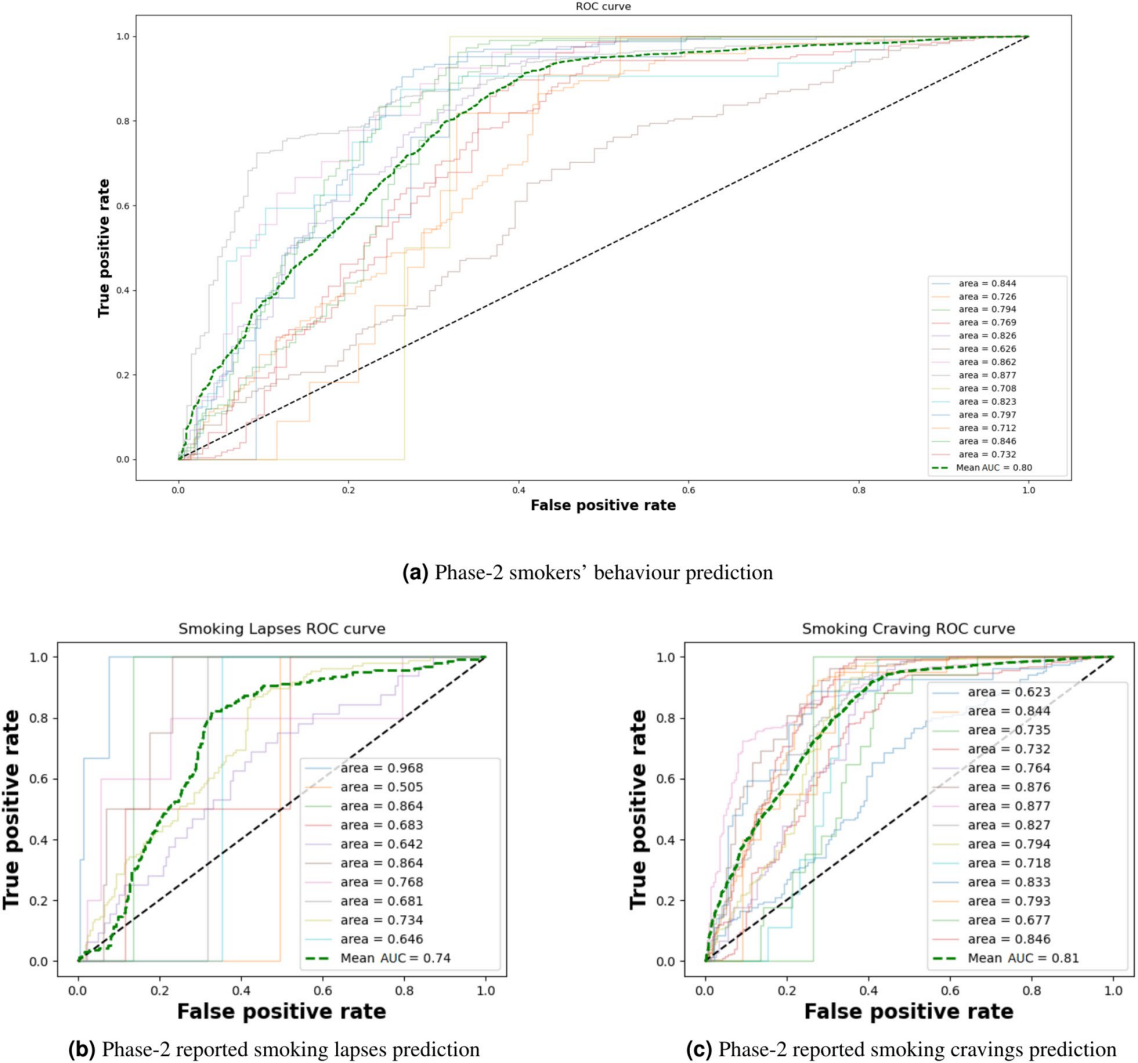
The 1D-CNN-BiLSTM ROC curve, trained using (**a**) Phase 1 data of 13 smokers, to predict the Phase 2 smoking patterns of the 14th smoker. (**b**) Predicting smoking events after re-classified Phase 2 data to separate smoking lapses and reported cravings and (**c**) Predicting craving reporting events. Note that the AUC value for smoking lapses is for only 10 participants, as the other 4 did not report lapses during the quit period. Light coloured lines represent each of the participants while the green dotted line represents the average of all participants.

Figure 4 shows the Receiver Operating Characteristic (ROC) curve for the model, which is a performance measure used in ML to understand the capability of a model to distinguish between different classes. While the Area Under the ROC Curve (AUC) in Fig. 4a values range between 0.68 to 0.87, the 1D-CNN-BiLSTM achieves an average AUC = 0.8 within a 5-minute window. The degradation in the model’s capacity to achieve accurate prediction for certain participants lacks a specific indicator, suggesting that there is no specific pattern of their behaviour that could help the model improve its prediction rate. In Fig. 4b and c labels were reclassified to separate smoking lapses and reported craving level, to enable the analysis of the ROC for both types separately. Figure 4 shows that the 1D-CNN-BiLSTM has overall good prediction, with higher ability to predict cravings (AUC = 0.81) than reported smoking lapses (AUC = 0.74).

## Discussion

The research has demonstrated that human movements, as detectable by smartphones’ sensors, can be used to predict health behaviours such as smoking lapses and cravings with higher accuracy than that achieved by human observable patterns (e.g. locations, time of day). Despite significant advances in the field of digital health and behavioural predictions and phenotyping, a notable gap exists in the processing of smartphone data using DL algorithms to develop effective characterisation, diagnostic and interventions tools for smoking-cessation and other health behaviours. In particular, the use of location and time of day are unlikely to be acceptable data sources if the intervention is intended to assist in cessation of habits involving illegal substances.

To date, applications that are designed to support smoking identification and/or support for cessation, if using ML algorithms at all, have primarily focused on motion sensors from wearable devices to identify specific actions (e.g. arm movement to identify smoking action^26–28^) or self-reported smoking triggers (e.g. locations^9^) neglecting the wealth of information that can be collected from the multifaceted sensors embedded in smartphones. There is therefore a need to explore how DL models can be used to effectively predict behaviours and capture temporal patterns, and accurately identify the precise time-window in which support or interventions are needed. Such support for smokers may be particularly valuable during the first few months after quitting^29,30^.

Overall, this research posed the null hypothesis that passively collected data from smartphones, analysed using a DL algorithm, will have no significant effect on the prediction of smoking behaviour. To test this hypothesis, the Friedman test^31^ was used to compare multiple classifiers with various data types: ACC, GYR, MAG, L, T, and a combined ACC-GYR-MAG input. In the ensuing analysis, each of the four DL models (LSTM, CNN, BiLSTM, and 1D-CNN-BiLSTM), were subjected to two significant levels, $\alpha$ = 0.05 and $\alpha$ = 0.01. The evaluation criterion was based on the generation of p-values, where a p-value below the chosen 0.01 signifies a statistically significant difference. To this end, the outcome of the Friedman test for all four DL models rejects the null hypothesis (Table 2). These findings show the substantial impact of passively collected smartphones’ sensor data on the disparities in performance of the evaluated DL models in predicting smoking events.

**Table 2:** Summary of Friedman test results.

Model	Q-statistic	Degrees of freedom	p-value
LSTM	27.489796	5.0	0.000046
CNN	15.826446	5.0	0.007357
BiLSTM	28.106996	5.0	0.000035
1D-CNN-BiLSTM	19.016736	5.0	0.001908

Despite the many advances achieved by this work, it is not without limitations. First, the collected data was geographically restricted to the United Kingdom. This limitation raises questions about the generalisability of the findings to diverse demographics and cultural contexts. As the model relies on movements for which it is not known if there are cultural or regional differences, it would be good to validate this with data from other cultures. This is of particular interest given that smoking triggers vary across different populations and regions. For example, alcohol consumption is only a trigger in cultures that consume alcohol. Moreover, while the study demonstrates the effectiveness of the DL models in predicting smoking behaviour within the studied population, further validation in a diverse populations (e.g. individuals with limited mobility) is necessary to ensure the robustness and applicability of the models in universally supporting smokers through the quitting process. Expanding the dataset to encompass diverse populations and providing validation within real-world applications is crucial for ensuring the robustness and generalisability in the complex landscape of providing effective and timely intervention for all smokers.

Additionally, the performance of all the models is constrained by the necessary reliance on self-reported labels (i.e. smoking events, craving levels or relapses), which introduces an unknown level of noise into the labels. As a result, aggressively optimising prediction accuracy may increase the risk of over-fitting. This limitation underscores the importance of exploring diverse model types, as robustness, in this case across models, becomes an important factor in assessing the handling of noisy ground truth data. While the use of self-reporting would not be needed if the model was to be implemented in an application, it was necessary for the training and validation of the model. To increase the reliability of self-reporting, the reporting process was made very simple (a single button-press on the app), and it occurred in real time (no recollection was needed). In evidence of the reliability of self-reporting in this dataset, there is overall consistency within each participant, such that smoking events are reported in similar time over the period of reporting. Future studies may wish to validate the model in real time, by letting smokers use the app, and confirm if predictions notifications are indeed accurate.

A further potential limitation of this study relates to the brief motor activity associated with self-reporting, which may suggest the model is predicting this action, and not smoking/craving events. However, this interaction represents only a minimal section of the 5-minute analysis window, reducing the likelihood that the model’s predictions are driven primarily by this short movement. Furthermore, reaching for the smartphone to log an event is likely to involve movement patterns similar to everyday actions such as reading a message or opening an application. Labelling each of these events as a smoking / craving event would significantly reduce the accuracy of the prediction (especially for craving, which may have only been reported once per day). Hence, while it could be a possible limitation, the study design, which analyses an extended temporal window rather than isolated moments, supports the interpretation that the model captures broader movement dynamics rather than singular device-interaction gestures.

Overall, the outcome of this research represents real advancement in modelling and predicting smoking behaviour. This is of particular importance given that the DL model was trained using data collected in an uncontrolled environment, with participants going about their usual daily routines without any changes except for pressing a button to report smoking events in real time during a 2-week pre-quit period, and reporting smoking lapses and craving during a 3-month post-quit period. These will likely not be needed when the model is implemented into a smoking cessation app, given the results show good prediction rate even when an individual’s data is not used.

For future deployment, it will be essential to ensure ethical compliance, including adherence to the EU AI Act (or its analogues in other jurisdictions), ensuring model transparency, expandability, and obtaining informed consent from users. With these restrictions, future work should explore to what extent data from these sensors can be used to predict or characterised other human behaviours that are not considered genetic or those that are specifically associated with visible changes in movement

## Methods

In order to develop an effective smoking-behaviour identification model, it was necessary to create a large dataset that includes smoking events, as reported in real time, alongside automatically collected data from the smokers’ smartphone sensors. This dataset can be made available upon request, adhering to ethical and commercialisation requirements. Using this dataset, the present study focused on the development of a DL model that could be trained to learn patterns within these data to predict smoking-related behaviour. The focus is exclusively on comparing advanced DL architectures, evaluating and comparing 1D-CNN, Long Short-Term Memory (LSTM), Bidirectional LSTM (BiLSTM), and hybrid combined models. This focus is driven by previous work, which demonstrated that DL approaches, particularly the 1D-CNN, outperformed traditional methods (such as Support Vector Machines: SVM and Decision Trees: DT models) in predicting smokers’ behaviour^13^.

### Creation of dataset

Seventeen smokers (10 Females and 7 males, Mean age 37.18, minimum 5 cigarettes a day for at least 6 months) were recruited for the study and were financially compensated for their time. The study received full ethical approval from the Manchester Metropolitan University ethics committee. Before condensing and cleaning the data, the entire dataset included 404478 5-min samples. These were divided based on the two phases of data-collection Phase 1, pre-quitting, included 81837 5-minute samples, while Phase 2, post-quitting, included 322641 samples. The final data set (after pre-processing, see Sect. "Data processing" below) used for the training and validation process included a total of 2572 Phase 1 and 3616 Phase 2 5-minute data period samples (overall smoking lapses: 158, craving reporting: 1650, and 4337 non-smoking events).

Data were collected using a simple smartphone app. In Phase 1 of the data collection smokers were asked to report in real time, by pressing a button on their app, every cigarette they smoked over a two-week period. At the end of the two weeks, participants were given a 5-day window in which to quit smoking, before progressing to the Phase 2 of data collection. In Phase 2, participants were asked to report, over the three-month post-quitting period, any smoking lapses. They were also asked to report their craving level on a scale from 1 (very low) to 5 (very high), every time that they experienced high craving, or at least once a day if they did not experience high cravings (whenever they remembered to do so). This was important as a measure of engagement with the app. Smartphones’ sensor data (Accelerometer: ACC; Gyroscope: GYR; Magnetometer: MAG; Light: L; Time of the Day: T and GPS) were continuously recorded every minute throughout both phases of data collection.

### Data processing

The collected data was pre-processed and then fed into four different DL models. Each model was assessed for its effectiveness in predicting smoking events during the pre-quit period (Phase 1) and instances of smoking lapses and cravings after quitting (Phase 2).

To develop a DL prediction model with a 5-minute prediction period, the collected data was converted to non-overlapped 5-minutes samples. This is common practice in action prediction ML models when training uses samples that are derived from raw signal data^32,33^. All data samples with a majority of missing samples were removed (as these are not useful for modelling), and the remaining samples were assigned either a (0) value for non-smoking samples or (1) for smoking events pre-quitting and reported cravings and lapses post quitting. Finally, to avoid the unbalanced data problem^34^, the data was randomly down sampled with noise removal^35,36^. Down sampling may be considered problematic in addiction research^37^ as the emphasis is on the ‘event’ (lapsing) rather than ‘non-event’ (non-smoking is a non-event); therefore additional results are reported which consider the non-sampled data (as processed through the current algorithm) in Appendix A. Each data sample included 25 rows of smartphone sensor data, with each row consisting of 5 columns (i.e. ACC, GYR, MAG, L, & T). GPS data was not used in the model, as due to the sensitivity of the data, modern smartphones only enable GPS data collection when the app is open. This corresponded with smokers reporting an event on the app, making the data highly biased as it was only available for smoking and craving samples.

The model can predict smoking events and cravings with high accuracy within a 5-min period. This represents enormous progress compared to previous work^13^. It is concluded that: (a) a combined 1D-CNN-BiLSTM is superior in computing temporal patterns of smoking behaviour, (b) combined accelerometers, gyroscopes, and magnetometers data give best predictions compared to each on their own, and compared to other predictors, and that (c) the model is capable of predicting the behaviour of new smokers, whose data was not used to train the DL model. The developed DL algorithm achieved 0.85 accuracy in predicting smoking behaviour before quitting and 0.77 accuracy in identifying high cravings and lapses post-quitting. This precision in predicting high-risk situations presents a promising opportunity for delivering timely interventions to support individuals during the quitting process. Despite some limitations, the findings underscore the promising role of smartphones’ sensors’ data and DL methods in advancing the understanding and intervention provision for smoking, and potentially other health behaviours, paving the way for more personalised and effective health interventions.

### Deep learning model for smoking behaviour prediction

The research utilised a stacked DL model that combines 1D-CNN^38^ and BiLSTM^39,40^ units. In the combined DL model, the 1D-CNN component can learn local patterns from the input data but it cannot learn sequential correlations, whereas the BiLSTM component is specialised for sequential modelling, and can extract correlated patterns. Therefore, the combined model has the potential to improve the prediction accuracy of smoking (and craving) events, as this requires processing of large environmental datasets produced by phone sensors (input), while correlating it with smoking related events in the past and the (sequential correlations). This approach was shown to be effective in several domains, including time series prediction and health applications^38,41,42^.

The input layer accepts *n*-input vector (*n* is the number of inputs that changes based on the used data, ACC, GYR, MAG, T, & L) each with 25 rows of data (at 5 samples of data for each minute, 25 rows represents a total duration of 5 minutes). This is passed to the convolutional layer, with a filter size of 128; the convolutional operation takes the form

1 $$\begin{aligned} C_i=h(w^T\bigotimes x_{i-10:i}\ +b_i), \end{aligned}$$

where $\bigotimes$ is convolution operator, $w^T$ the network weights, *b* is the bias and *h* is a non-linear ReLU activation function with $l_2$ weight regularisation. The feature map output of this layer is then batch normalised^43^, followed by a max-pooling layer that reduces the features’ variance,

2 $$\begin{aligned} P_{i,k}\ =\ Max\ (C_{(i,k)\ }U_{3,1}), \end{aligned}$$

where *k* is the filter number, and $U_{3,1}$ is sliding max window of size (3 x 1).

The second stage of the DL model is the BiLSTM network^43,44^ (combining forward and backward LSTM models). Each LSTM consists of several multiplicative memory cells, each with three gates: input, output, and forget. These gates control the output from the cell, which is either ‘keep’, ‘release’, or ‘reset’; $c_t$ denotes the cell state (memory state) at time step *t*, which carries long-term information through the sequence, and $h_t$ denotes the hidden state at time step *t*, which represents the output of the LSTM cell and is passed to the subsequent layers.

3 $$\begin{aligned} f_t^\rightarrow= & \ \sigma \left( w_f^\rightarrow \cdot \left[ h_{t-1}^\rightarrow ,\ x_t^\rightarrow \right] +b_t^\rightarrow \right), \end{aligned}$$

4 $$\begin{aligned} i_t^\rightarrow= & \ \sigma \left( w_i^\rightarrow \cdot \left[ h_{t-1}^\rightarrow ,\ x_t^\rightarrow \right] +b_i^\rightarrow \right), \end{aligned}$$

5 $$\begin{aligned} {\widetilde{c}}_t^\rightarrow= & \ tanh{\left( w_c^\rightarrow \cdot \left[ h_{t-1}^\rightarrow ,\ x_t^\rightarrow \right] +b_c^\rightarrow \right), } \end{aligned}$$

6 $$\begin{aligned} c_t^\rightarrow= & \ f_t^\rightarrow *{\widetilde{c}}_{t-1}^\rightarrow +i_t^\rightarrow *{\widetilde{c}}_t^\rightarrow, \end{aligned}$$

7 $$\begin{aligned} o_t^\rightarrow= & \ \sigma \left( w_o^\rightarrow \cdot \left[ h_{t-1}^\rightarrow ,\ x_t^\rightarrow \right] +b_o^\rightarrow \right), \end{aligned}$$

8 $$\begin{aligned} h_t^\rightarrow= & \ o_t^\rightarrow *t a n h{\left( c_t^\rightarrow \right) }. \end{aligned}$$

In the BiLSTM $f_i$, $i_t$, and $o_t$ are calculated for both forward and backward networks; $h_t^\rightarrow$ for the forward LSTM network is calculated using the current *t* and the previous $t-1$ instances, While the $h_t^\leftarrow$ for the backward LSTM network is calculated using the current *t* and the next $t+1$ as

9 $$\begin{aligned} f_t^\leftarrow= & \ \sigma \left( W_f^\leftarrow \cdot \left[ h_{t+1}^\leftarrow ,\ X_t^\leftarrow \right] +b_t^\leftarrow \right), \end{aligned}$$

10 $$\begin{aligned} i_t^\leftarrow= & \ \sigma \left( W_i^\leftarrow \cdot \left[ h_{t+1}^\leftarrow ,\ X_t^\leftarrow \right] +b_i^\leftarrow \right), \end{aligned}$$

11 $$\begin{aligned} {\widetilde{c}}_t^\leftarrow= & \ tanh{\left( W_c^\leftarrow \cdot \left[ h_{t+1}^\leftarrow ,\ X_t^\leftarrow \right] +b_c^\leftarrow \right) }, \end{aligned}$$

12 $$\begin{aligned} c_t^\leftarrow= & \ f_t^\leftarrow *{\widetilde{c}}_{t+1}^\leftarrow +i_t^\leftarrow *{\widetilde{c}}_t^\leftarrow, \end{aligned}$$

13 $$\begin{aligned} o_t^\leftarrow= & \ \sigma \left( W_o^\leftarrow \cdot \left[ h_{t+1}^\leftarrow ,\ X_t^\leftarrow \right] +b_o^\leftarrow \right), \end{aligned}$$

14 $$\begin{aligned} h_t^\leftarrow= & \ o_t^\leftarrow *t a n h{\left( c_t^\leftarrow \right) }. \end{aligned}$$

The final $h_t$ is the concatenated vector of both $h_t^\rightarrow$ and $h_t^\leftarrow$,

15 $$\begin{aligned} h_t\ =[h_t^\rightarrow \ h_t^\leftarrow ]. \end{aligned}$$

The output from the BiLSTM is then passed to the final level of the model, which is made of three layers of a fully connected neural network. Specifically, the concatenated hidden state $h_t$ is used as the input to the first dense layer. These are used to generate a binary output that represents a prediction of either a smoking or a non-smoking session in a 5-minute slot.

## Conclusion

The research findings presented here offer insight into the untapped potential of processing human movements, as detected by digital sensors, through DL algorithms in order to predict seemingly unrelated behaviour. This is demonstrated by showing the high reliability of the model in predicting smoking behaviour before and after quitting.

This state-of-the-art work can predict smoking events and cravings with high accuracy within a 5-min period. This representing a substantial progress in comparison to our to our previous work^13^. It is concluded that, (a) a combined 1D-CNN-BiLSTM is superior in computing temporal patterns of smoking behaviour (b) combined accelerometers, gyroscopes, and magnetometers data give best predictions compared to each on their own, and compared to other predictors, and that (c) the model is capable of predicting the behaviour of new smokers, whose data was not used to train the DL model. The proposed DL model achieved 0.85 accuracy in predicting smoking behaviour before quitting and 0.77 accuracy in identifying cravings and lapses post-quitting. This precision in predicting high-risk situations presents a promising opportunity for delivering timely interventions to support individuals during the quitting process. Despite some limitations, the findings underscore the promising role of smartphones’ sensors’ data and DL methods in advancing the understanding and intervention provision for smoking, and potentially other health behaviours, paving the way for more personalised and effective health interventions.

## Data Availability

The data supporting the findings of this study are available from the authors upon request from Dr. Maryam Abo-Tabik, “mabo-tabik@lancashire.ac.uk”. Access will be granted free of charge for research and educational purposes. For commercial applications, a fee may apply. A data sharing agreement will be required to ensure appropriate use, attribution, and compliance with ethical standards. Users are expected to cite this work in any resulting publications or derivative projects. The code can be accessed via GitHub Repository.

## A. Effects of prevalence manipulation

The performance of the model, predicting either smoking or craving on the test data as report in the body of the paper is described in Tables  3 and 4 follows:

**Table 3:** Confusion matrix for smoking/craving prediction.

	Real smoking/craving	Real non-instance	Total
Pred smoking/craving	1266	271	1537
Pred non-instance	542	1537	2079
Total	1808	1808	3616

**Table 4:** Performance metrics.

Metric	Value
Sensitivity (recall)	0.70
Specificity	0.85
PPV (precision)	0.82
NPV	0.74
Prevalence	0.50
Accuracy	0.78

It should be noted that the prevalence (the fraction of instances that are real smoking/craving events) is 0.5; this is a consequence of re-weighting the data, to reduce the number of real non-instance events. This is typically done in order to reduce the bias in prediction. It is therefore important to explore whether the high prediction rate presented in the article is affected by the procedure of unbiasing the data. The equivalent measures for the same events without the re-weighting are given in Tables 5 and 6.

**Table 5:** Confusion matrix for smoking/craving prediction.

	Real smoking/craving	Real non-instance	Total
Pred smoking/craving	1266	651	1917
Pred non-instance	542	3686	4228
Total	1808	4337	6145

**Table 6:** Performance metrics.

Metric	Value
Sensitivity (recall)	0.70
Specificity	0.85
PPV (precision)	0.66
NPV	0.87
Prevalence	0.29
Accuracy	0.81

Table 6 reveals that the prevalence of smoking / craving events is now 0.29, as there are many more non-smoking / non-craving instances than smoking / craving instances (as one expects in a real life situation). However, the accuracy of prediction of smoking / craving events is slightly higher than the one reported in Table 4, while the sensitivity and recall are unaffected. The change in accuracy affects the drop in Positive Predictive Value (PPV, how often a smoking event is predicted accurately) and the associated rise in Negative Predictive Value (NPV, how often a non-smoking event is predicted accurately). However, the overall performance is not affected by the change.

Individuals vary in their smoking / craving frequency; this alters the prevalence of smoking/ craving events in their data. When building an individual model, it might be appropriate to include this information (generally as a bias within the final thresholding operation classifying instances as smoking / craving vs. a non-instance). However, this is not appropriate in this case, as the general model presented here is designed to provide a generic way to support any smoker. Including the individual prevalence of positive events at either the training or testing stage could have the effect of over-fitting the model. While the choice of a prevalence of 0.5 is arbitrary it allows for each test instance to be considered blind, without reference to other events or their expected category (e.g. smoking / craving vs. null). Future work may wish to examine if a different arbitrary threshold (e.g. 60:40) of non-smoking to smoking events may be more suitable for prediction of smoking behaviour.

To further examine model performance under extreme class imbalance, the expected performance was evaluated at a prevalence of 1%, while preserving sensitivity and specificity. The corresponding confusion matrix and performance metrics are shown in Tables 7 and 8.

**Table 7:** Confusion matrix for smoking/craving prediction (1% prevalence).

	Real smoking/craving	Real non-instance	Total
Pred smoking/craving	31	651	682
Pred non-instance	13	3686	3699
Total	44	4337	4381

**Table 8:** Performance metrics (1% prevalence).

Metric	Value
Sensitivity (recall)	0.70
Specificity	0.85
PPV (precision)	0.045
NPV	0.996
Prevalence	0.01
Accuracy	0.85

As would be expected, sensitivity, and specificity remain unchanged (the slight alteration reflects quantisation of the small number of positive cases), since these metrics are independent of prevalence. PPV decreases substantially under low prevalence, while NPV increases, reflecting the natural dependence of predictive values on base rates rather than any loss of the model’s ability to discriminate events. It should be noted that training directly on extremely unbalanced data of this type would be unrealistic, as the model would be biased toward the majority class and unable to learn rare-event patterns effectively.

In this study the model has not been optimised for population-wide screening at very low prevalence, nor have thresholds been tuned to reduce false positives. This study is a proof-of-concept demonstrating discriminative feasibility in a generic setting. In practical deployment, precision could be improved through threshold calibration, cost-sensitive optimisation, or focusing on higher-risk subgroups. Thus, the low PPV at 1% prevalence reflects the deployment context, not a limitation of the underlying classifier.
